# Bio-mimetic synthesis of catalytically active nano-silver using *Bos taurus* (A-2) urine

**DOI:** 10.1038/s41598-021-96335-2

**Published:** 2021-08-20

**Authors:** Prashant D. Sarvalkar, Rutuja R. Mandavkar, Mansingraj S. Nimbalkar, Kiran K. Sharma, Pramod S. Patil, Ganesh S. Kamble, Neeraj R. Prasad

**Affiliations:** 1 School of Nanoscience and Technology, Shivaji University Kolhapur, Kolhapur, 416004 India; 2 Department of Botany, Shivaji University Kolhapur, Kolhapur, 416004 India; 3 Department of Engineering Chemistry, Kolhapur Institute of Technology’s College of Engineering (Autonomous), Affiliated to Shivaji University Kolhapur, Kolhapur, 416234 India

**Keywords:** Biotechnology, Materials science

## Abstract

Herein we have synthesized silver nanoparticles (Ag NPs) using liquid metabolic waste of *Bos taurus* (A-2 type) urine. Various bio-molecules present in cow urine, are effectively used to reduce silver (Ag) ions into silver nanoparticles in one step. This is bio-inspired electron transfer to Ag ion for the formation of base Ag metal and is fairly prompt and facile. These nanoparticles act as a positive catalyst for various organic transformation reactions. The structural, morphological, and optical properties of the as-synthesized Ag NPs are widely characterized by X-ray diffraction spectroscopy, ultraviolet–visible spectroscopy, scanning electron microscope, Fourier transmission infra-red spectroscopy, and atomic force microscopy. The as-synthesized bio-mimetic Ag NPs show potential activity for several reduction reactions of nitro groups. The Ag NPs were also used for degradation of hazardous dyes such as Methylene blue and Crystal violet with good degradation rate constant.

## Introduction

Since the dawn of civilization on the planet, material scientists are actively involved in fabrication of new materials with desired novel properties^1^. Also various type of physical treatment can affect the properties of the materials such as earthen pot after heating beyond a particular temperature becomes porous and does not get dissolved in water^2^. Thus the use of innovative material is the mirror of developmental scenario of human civilization and therefore particular age is many times named after the materials in use such as iron age, plastic age and now stepped into nano age^3,4^. Now the scientists working in the domain of material science are mainly interested to develop materials at the nano-scale^5–14^. Due to alien properties nanoparticles find wide range of applications in diverse areas such as in fabrication of various types of sensing devices such as solar cells, electrochromic materials, gas sensors, bio-sensors such as blood glucose sensors^15,16^, oximeter^17^, memristors^18^, meta-materials^19^ which have negative refractive indexes, energy storage devices such as supercapacitors using transition metal oxides, bio-active materials such as anti-microbial and anti-neoplastic agents^20,21^, heterogeneous catalyst for organic transformation and organic synthesis reactions, heterogeneous catalyst for dye degradation reactions and several types of electronic devices etc. The exotic properties developed in materials at nano-scale is due to various factors such as enlarged surface area which significantly increases active sites for reaction^22^, insignificant gravitational force, possible development of quantum effect, sensitive coulomb's force of attraction or repulsion, alive dangling bonds, constructive random molecular motion, remarkable surface tension and secondary bonds like Van der Waal's attraction, etc. These properties make nano-materials different from their bulk counterparts^23–27^. The physical and chemical properties of bulk material are supposed to be constant irrespective of size and weight of material under consideration such as the melting point of metal or refractive index of a liquid are having fixed value. These well-defined physical and chemical properties of the bulk materials reveal interesting properties at nano-scale. But due to exceptional difference in properties at nano-scale from that of their bulk counterparts many scientists believe the nano regime as a separate state of matter. In fact, the properties of bulk material are an average of the properties at the nano regime.

Our research group has successfully synthesized some transition metal and metal oxide nanoparticles such as Cd, CuO and Pd nanoparticles using Indian cow urine. In the current experimentation process, we have successfully synthesized Ag nanoparticles using A-2 type cow urine. *Ayurveda* is an ancient system of natural and holistic medicine developed particularly in Indian sub-continent. *Ayurveda* literature describes the use of cow products for medicinal purpo ses. Liquid metabolic waste of cow is a constituent of *Panchagavya* (a combination of cow urine, milk, clarified butter, curd and dung). According to description in classical *Ayurvedic* literature, the cow urine have been found to have approximately water 95%, Urea 2.5%, and the rest 2.5% is a mixture of different minerals, salts, hormones, and enzymes^28^. According to ancient Indian *Ayurvedic* literature liquid metabolic waste of cow is useful to control various ailments especially chronic diseases such as seizer disease, skin disease, hepatic diseases, psoriasis, paralysis, thyroid dis-order, constipation, abdominal diseases, renal disorders, diabetes mellitus, anticonvulsant drug etc. Some medical professionals claims that cow urine is an effective anti-neoplastic agent^28^. Cow urine is found to have beneficial properties particularly in the area of agriculture and therapeutics. It has been observed that the urine of Indian cow is highly effective and interestingly almost nil or very few medical properties are present in urine of crossbred, exotic cows, buffaloes etc. Recent researches showed that cow urine enhances immune status of individual through activating the macrophases and augmenting their engulfment power as well as bactericidal activity.

## Laboratory synthesis of nanoparticles

The nanoparticles can be categorized as (1) natural nanomaterials, (2) incidental nanomaterials and (3) engineered nanomaterials. Nano-particles possess wonderful exotic properties. Because of continuous requirement of nanoparticles in various appliances researchers intensely synthesized materials of various size and shape which are known as engineered nanomaterials. Due to simplicity, comfortable set up, economical consideration and defect free product bottom-up approach is becoming popular and now a day widely adopted. The bottom up route of synthesis implies that the nanostructures are synthesized by stacking atoms onto each other. This gives rise to crystal planes, crystal planes further stack onto one another which results in the formation of nanostructures. Thus bottom up approach can be simply viewed as synthetic route where the building blocks are added to have a nanostructure^11,23,29–31^.

## Materials and methods

### Bio-mimetic synthesis of Ag NPs using *Gir* cow urine

Ag NPs were synthesized using the liquid metabolic waste of indigenous Indian healthy *Gir* (A-2) cow of age approximately 7 years. The cow was regularly vaccinated by a veterinarian against common livestock diseases like rinderpest and black quarter etc. The cow urine is procured with agreement of animal rearer from cattle farm belonging to village Kaneri, District Kolhapur, India. The freshly discharged cow urine was collected in a sterile screw-capped bottle and brought in laboratory. The liquid metabolic waste of *Gir* cow was dribbled using filter paper and stored in suitable container at room temperature.

For the experimentation process, analytical grade silver nitrate precursor was purchased. Here 100 mL of 0.1 M AgNO_3_ solution was prepared by dissolving silver nitrate in double-distilled water. Then 15 mL 0.1% w/v cetyltrimethylammonium bromide (CTAB) a cationic surfactant was slowly added in above solution with constant stirring. The borosilicate glass made burette was filled with cow urine and drop wise added in silver nitrate solution with constant stirring. The reaction mixture was maintained in the range of 25–30 °C temperature. The addition of cow urine results in the formation of dark blackish colored precipitate of Ag in nano form. When 25 mL of cow urine added there was formation of sufficient amount of precipitate. The colloidal solution was continuously heated till complete evaporation of water content takes place. The, as-synthesized Ag NPs was annealed at 495 °C for 1 h. Then, the black-grey colored accumulated solid mass was separated using a metallic spatula and then it has been crushed mechanically into fine powder. Finally, as-synthesized Ag NPs were used for further characterization and catalytic reactions^5,25,26^.

### Possible reaction mechanism

**Figure 1:**
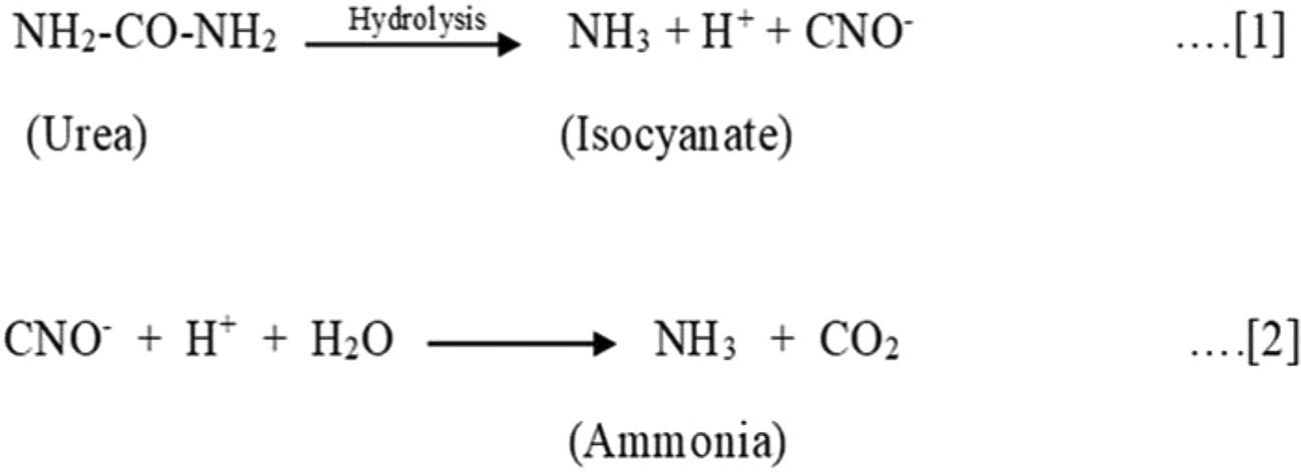
Conversion of urea to ammonia by hydrolysis.

The chemical formula for urea is CO(NH_2_)_2_. Here two –NH_2_ groups are directly attached to carbonyl group i.e. C=O. Actually it looks that urea should be a base due to presence of lone pair of electron on nitrogen atom. But due to electronegative nature of carbonyl group, it becomes a neutral compound. However, when urea gets reacted in presence of enzyme urease or at high temperature then there is conversion of urea into ammonia by hydrolysis as shown in Fig. 1.

In first step of reaction, there is break down of Urea into Ammonia and isocyanate ions as byproduct. This reaction is reversible at pH less than 5 and greater than 12. In second reaction, Isocyanate get hydrolyze to produce Ammonia and carbon dioxide is evolved as a byproduct. Urea hydrolysis is higher at temperature 35 °C than at 15 °C. The effect of pH is only observed between the pH 6 and pH 8^32^.

### Spectral characterization

The biosynthesized AgNPs with the cow urine were characterized by using XRD (Bruker Ltd. Germany Model: D2 Phaser, Copper target = 1.54 Å), The optical absorbance of the AgNPs was recorded at wave length range 200–700 nm by using UV–visible spectrophotometer (Shimandzu, Model: UV-1800), morphology and the particle size of the as-prepared AgNPs were investigated by Scanning Electron Microscopic (JEOL Ltd. Japan Model: JSM-6360), Atomic force microscope (USA Model: INOVVA 1B3BE), Photoluminescence (HORIBA Instruments Model: Fluoromax-4). The degree of crystallinity, defects and disorders, particle size of AgNPs was determined by FT-IR spectra was carried out by JASCO Japan Model: FT/IR-4700. The particle size analysis and Zeta potential measurement experiments were carried out by Horiba SZ-100 nanoparticle analyzer.

## Ramification and discourse

The synthesized nano-particles were characterized using advanced spectroscopic and microscopic techniques. The electron microscopic analysis reveals the morphologies for synthesized nanoparticles.

**Figure 2:**
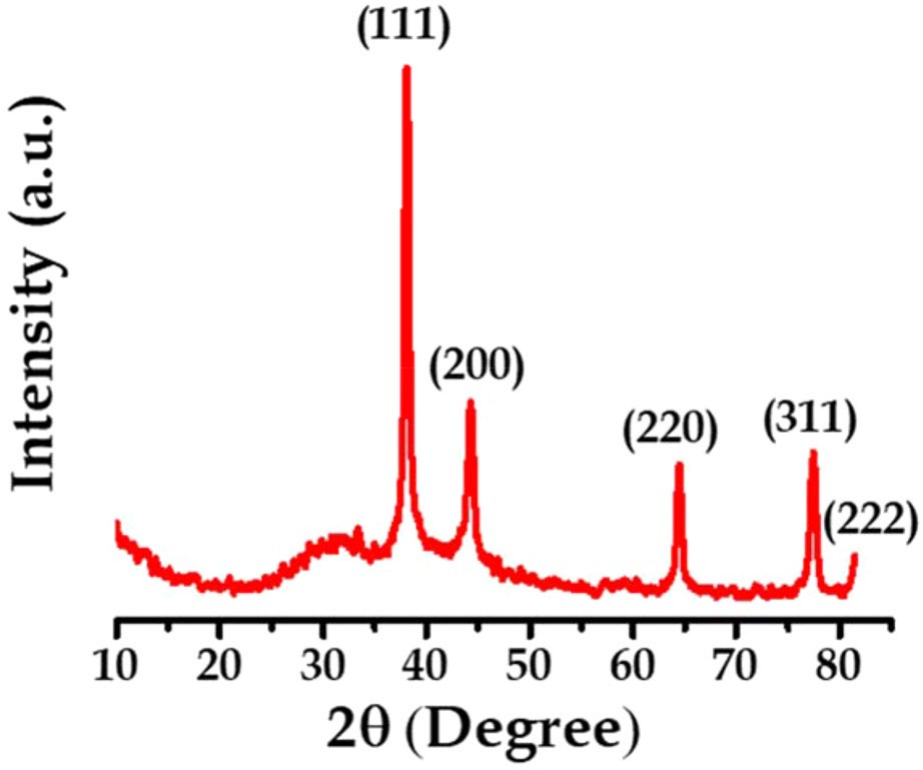
XRD pattern of Ag nanoparticle.

Solid state of materials can be further divided into two types such as crystalline forms and amorphous forms. XRD analysis is applicable to crystalline form of materials only. The phase pattern of AgNPs were characterized by XRD measurement. The XRD pattern reveals formation of poly-dispersed crystalline nano-material. The XRD pattern of biologically synthesized AgNPs is shown in Fig. 2. In XRD pattern, Bragg’s reflections are observed at 2θ values of 38.11, 44.19, 64.43, 77.38 and 81.53 representing (111), (200), (220), (311) and (222) planes, respectively; which indicates that AgNPs were nanocrystals with cubic face centred (FCC) structure. The peaks in the XRD pattern obtained confirmed that the biosynthesized material was pure AgNPs with highly crystalline nature. The patterns were consistent and are in agreement with the JCPDS card No. 00-003-0931.

The crystallite size can be calculated famous using Debye–Scherer’s formula^33–35^.

1 $$D= \frac{0.9 \uplambda}{{\beta cos} \uptheta}$$

The crystallite size is calculated using above formula and the average size of Ag NPs is 29.92‬ nm.

We are pretty familiar with the ever increasing demand of Ag nanoparticles in various industries. This is because of novel physical, chemical and biological properties developed at nano level. The specific surface area of a nanoparticle depends upon the interrelationship between the particle size and morphology. MI is calculated from FWHM of XRD to explore this relationship, based on earlier report ^35^.

MI is obtained using the equation,

2 $$\text{MI}=\frac{\text{FWHMh}}{\text{FWHMh }+\text{ FWHMp}}$$

where, M.I. is morphology index and FWHM_h_ is highest FWHM value obtained from peaks.

**Figure 3:**
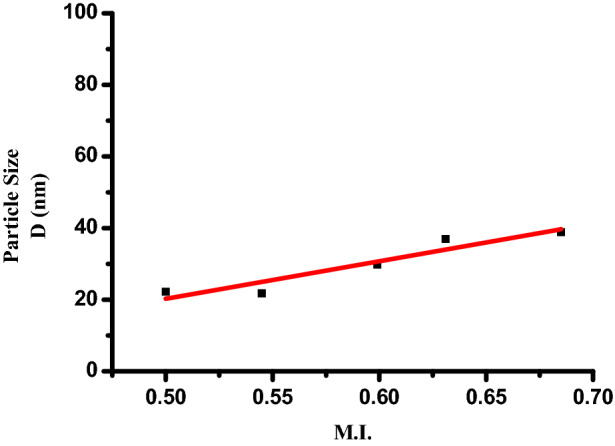
Morphological index vs particle size of Ag nanoparticle.

**Figure 4:**
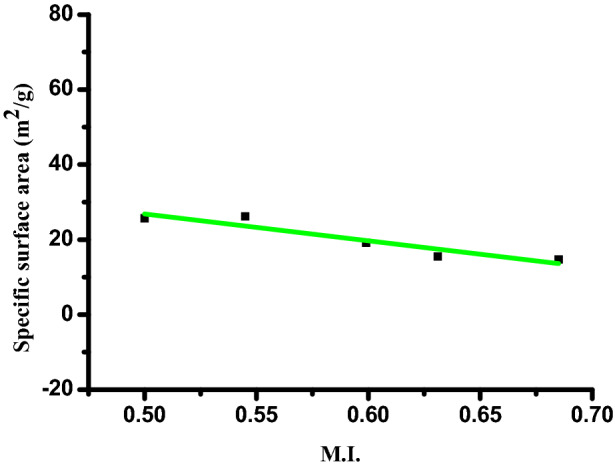
Morphological index vs specific surface area of Ag nanoparticle.

MI range of experimental AgNPs ranges from 0.50 to 0.685 and the details are presented in Table 1. It is correlated with the particle size (range from 38.84 to 21.79 nm) and specific surface area (range from 14.71 to 26.22 m^2^ g^−1^). From the calculated data it is observed that MI is directly proportional to particle size and inversely proportional to specific surface area with a small deviation. The results are shown in Figs. 3 and 4. Linear fit in the figures indicates the deviations and relationships between them.

**Table 1:** Analysis and calculation of various parameters.

Sr. no.	Peak position: 2θ (degree)	Full width half maxima (Å)	Full width half maxima (radians)	Particle size D (nm)	d-Spacing (Å)	For Ag unit cell edges: a = b = c (Å)	Specific surface area (m^2^ g^−1^)	Morphological indexing
1	38.11	0.2165	0.003778	38.84	2.3592	4.0857	14.71237	0.685
2	44.19	0.3936	0.006869	21.79	2.0481		26.22435	0.545
3	64.43	0.3149	0.005496	29.82	1.4449		19.16259	0.599
4	77.38	0.2755	0.004808	36.95	1.1406		15.46491	0.631
5	81.53	0.4723	0.008243	22.21	1.1796		25.72844	0.500

**Figure 5:**
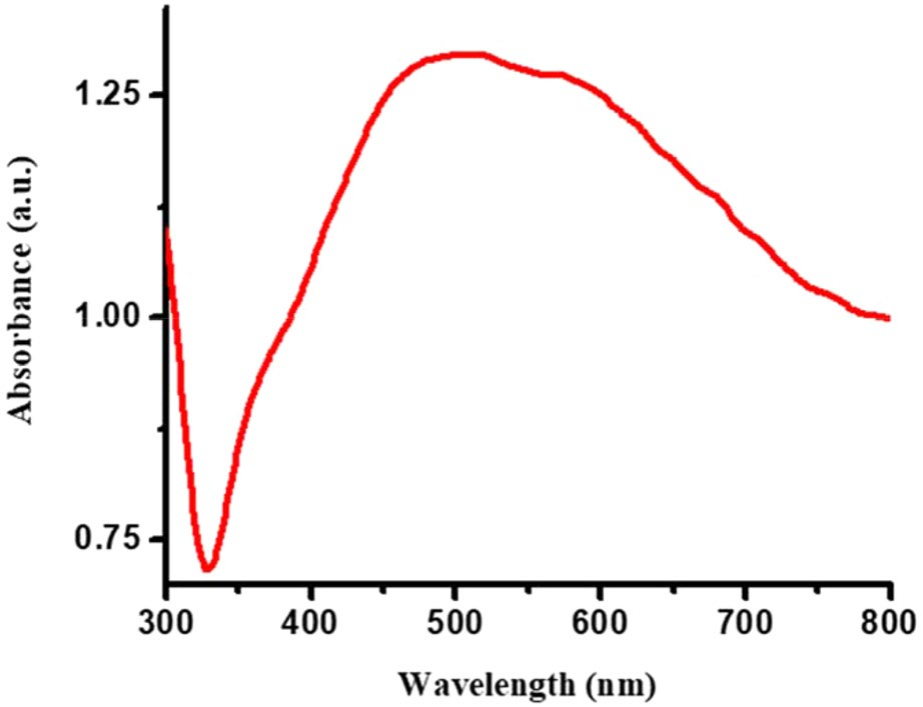
UV–Vis spectrum of Ag nanoparticle.

The insoluble black colored power obtained at the end of reaction was sonicated in bath sonicator so as to get well dispersed solution. UV–visible spectroscopic analysis (Fig. 5) shows maximum absorption is obtained at 500 nm which indicates that, the synthesized AgNPs nanoparticles efficiently absorb visible light therefore it could be acted photocatalyst for dye degradation in solar light. The absorption value i.e. redshift in λ_max_ value may be due to agglomeration of nanoparticles. This UV–Vis spectrum confirmed that the as-prepared AgNPs have been proficiently acted under visible light treatment so it could be also acted in presence of solar energy with more efficiently.

Scanning electron microscopy is a powerful tool for sample imaging with nanoscale magnification and resolution which visualizes very small topographic details on the surface of object. Synthesis of AgNPs using biological route (either plant extract or animal waste) shows relatively spherical in shape.

**Figure 6:**
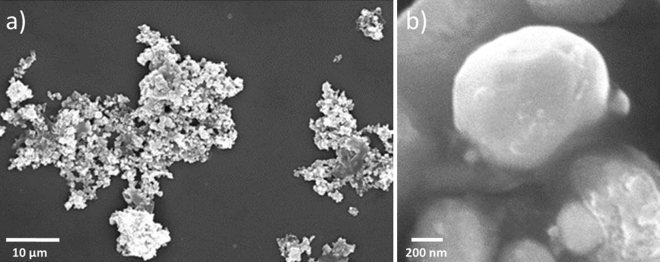
SEM image of Ag nanoparticle.

Herein, the morphology and the particle size of the as-prepared NPs were investigated by Scanning Electron Microscopic (SEM) analysis using MIRA3 TESCAN SEM machine and it is observed that Ag nanoparticles are in unique architectures with relatively spherical in shape (Fig. 6a). The mean diameter of AgNPs are approximately 90–200 nm as shown in Fig. 6b. From the structural point of view, SEM images reveal that homogeneous spherical shape of NPs have been formed. Since photocatalysis is a surface phenomenon activity and the homogeneous spherical shape of NPs could help to enhance the photocatalytic activities such as dye degradation and organic transformation reaction.

**Figure 7:**
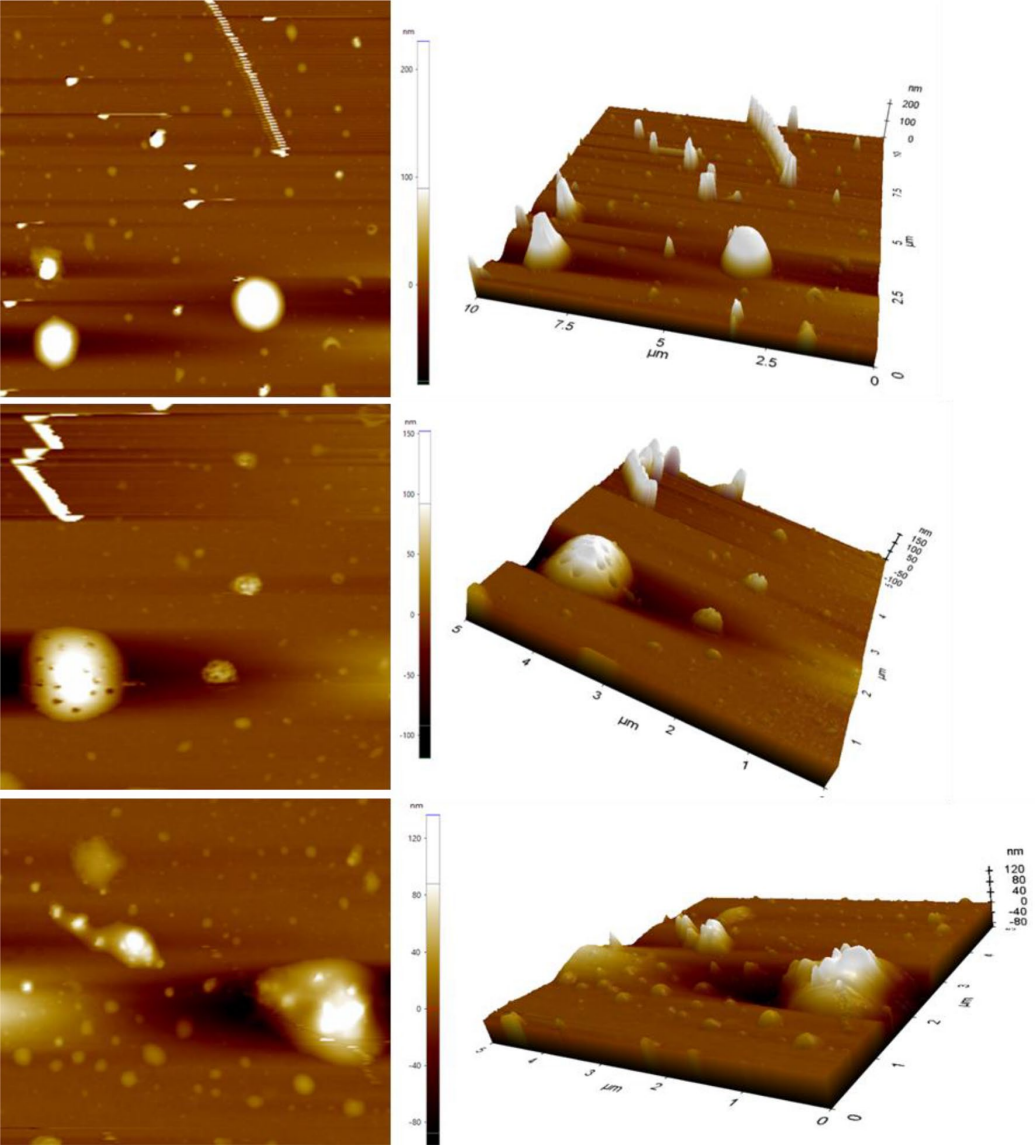
AFM 2D and 3D images of Ag nanoparticle.

The Atomic force microscope (AFM) is the most commonly used form of Scanning probe microscope. Atomic force microscopy is a three dimensional topographic technique with a high atomic resolutions and measures surface roughness. The attractive/repulsive forces between the sample surface and a sharp probe of NPS were detected by AFM and the force was dignified through a laser photodiode system that detects the difference in voltages at the photodetector output. The surface defects of AgNPs were characterized by AFM and it is shown in Fig. 7. In this study of AFM, the morphology of synthesized sliver nanoparticles was found to be highly spherical shape. The surface topography of AgNPs thin films observed that in two dimensional and three dimensional views at nanoscale^36,37^. It is clearly depicted that the as-synthesized AgNPs are spherical shape with homogeneous good thickness.

**Figure 8:**
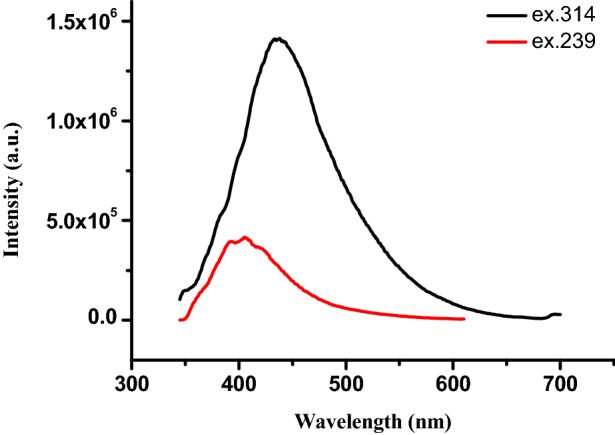
PL spectra of Ag nanoparticle (excitation wavelength 239 and 314 nm).

Photoluminescence is the phenomenon of emission of light from any type of matter when photon is incident on it. Photoluminescence (PL) is the tool to confirm the structural defect related properties. Figure 8 shows the PL spectra of Ag nanoparticles. PL spectra is consisting of two excitation peaks at 405 nm and 436 nm. Aqueous Ag NPs solution are showing peak at 405 nm with a excitation wavelength 239 nm and further peak come at 436 nm with a excitation wavelength 314 nm^38–40^.

**Figure 9:**
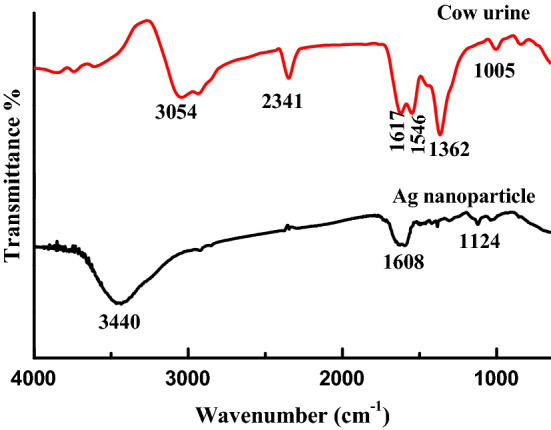
FT-IR spectra of cow urine and Ag nanoparticle.

FT-IR spectra show absorption bands that enable to determine the presence of various functional groups in a molecule. Various functional groups correspond to different frequencies and hence different wave numbers. In Fig. 9, a very strong and broad absorption band was found at 3440 cm^−1^ which indicates that the synthesized samples can have vibrations of symmetric stretching due to presence of primary amine. The other bands are observed at 1608 and 1124 cm^−1^ which may be due to aromatic stretch of organic compound present in synthesized nanomaterials. FT-IR spectra are the fingerprint due to presence of functional groups in the material under study. Figure 9 shows FT-IR spectra of *Bos taurus* (A-2 type) cow urine, the observed major peak positions are 3054 cm^−1^, 1617 cm^−1^, 1362 cm^−1^, which may be due to C–H aromatic stretching, 1617 cm^−1^ C–C aromatic stretching, 1362 cm^−1^ C=O stretching (ketonic group) respectively. Further Fig. 9 shows peak position at 3440 cm^−1^, 1608 cm^−1^, 1124 cm^−1^, showed a red shift. This may be due to interaction of Ag nanoparticle with function groups viz. C–H and AgNPs interaction, C–C and AgNPs interaction.

**Figure 10:**
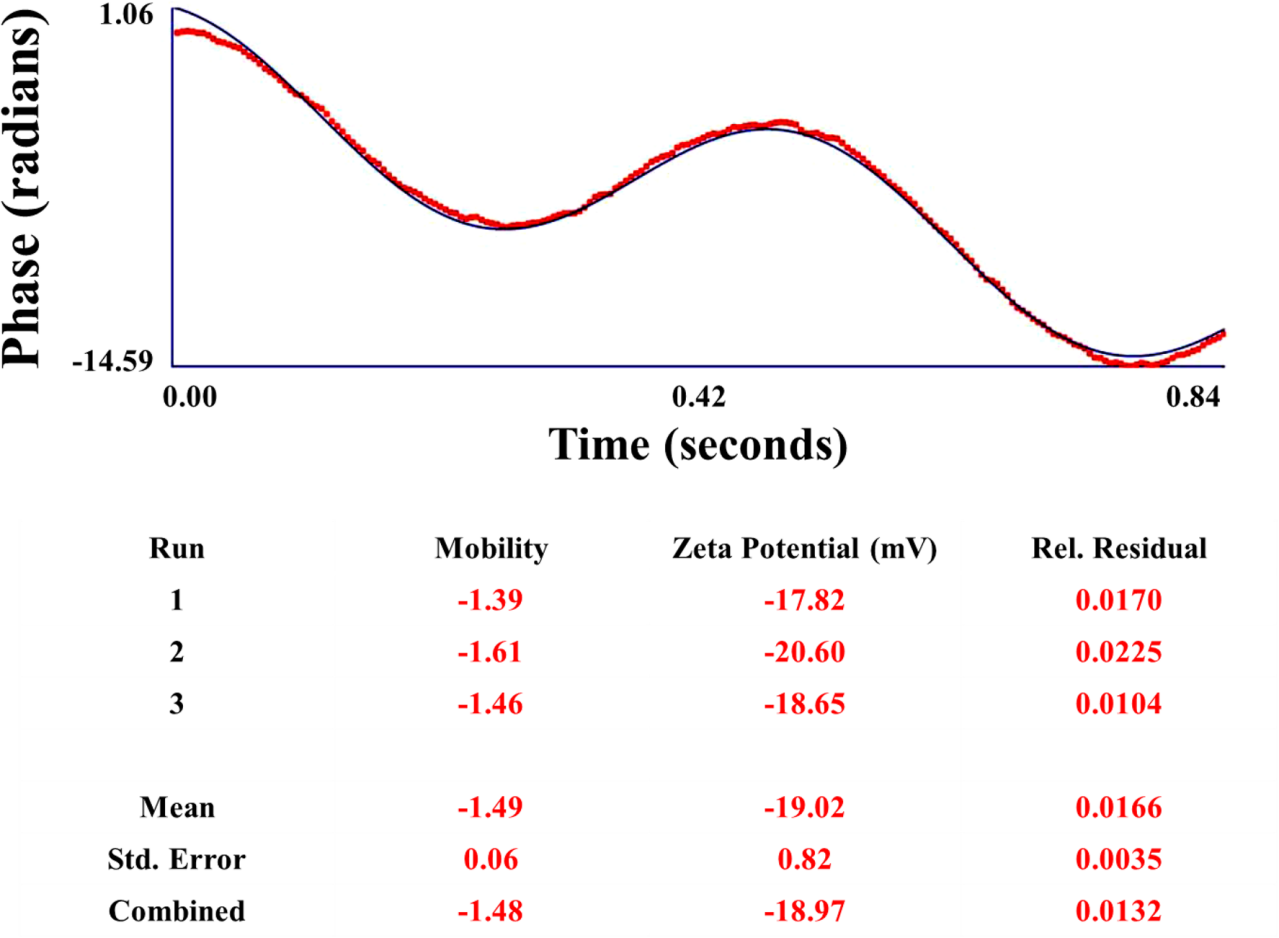
Zeta potential of Ag nanoparticle.

**Figure 11:**
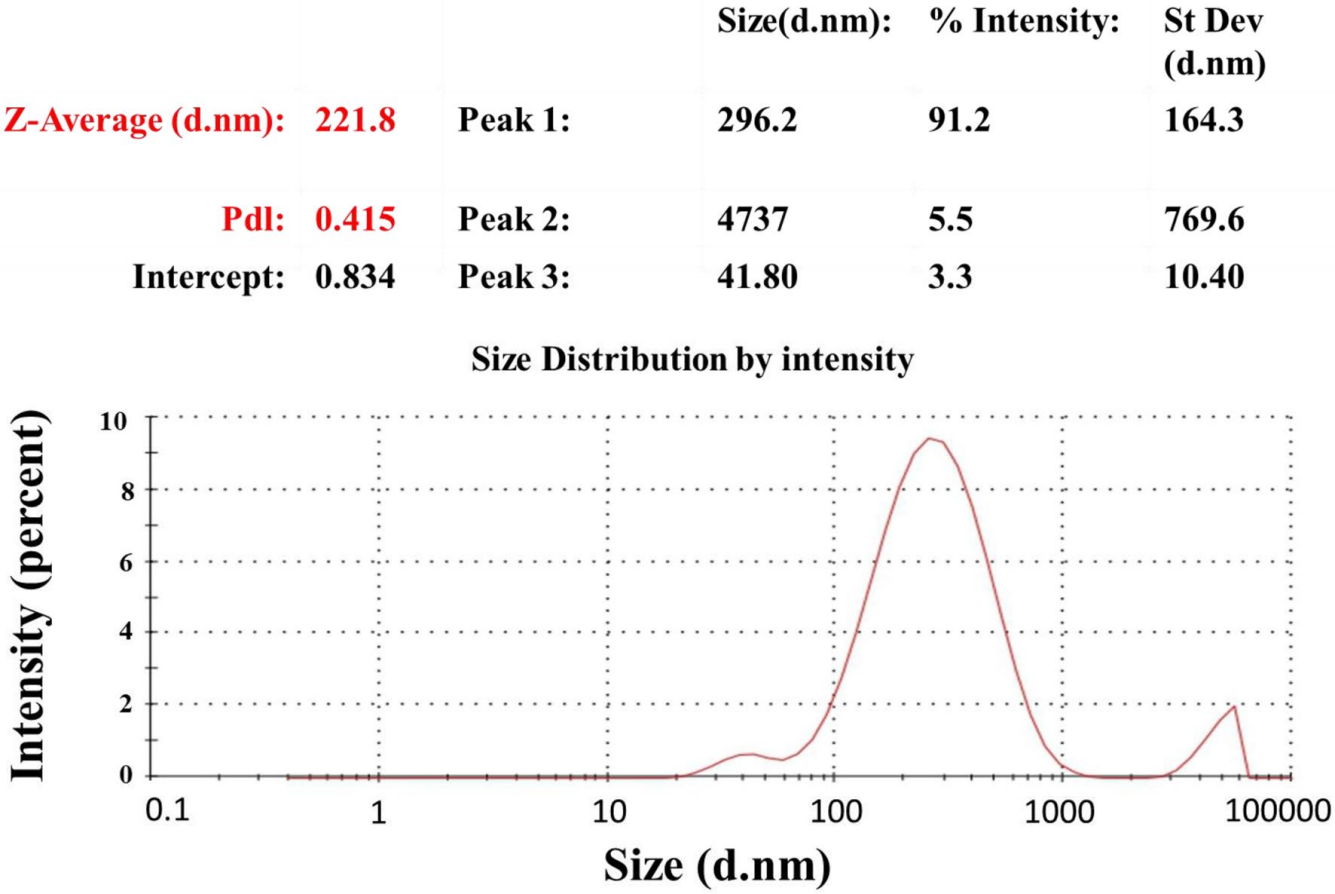
Dynamic light scattering of Ag nanoparticle.

Electro kinetic potential from Fig. 10 signifies the stability of the synthesized nanoparticles. The hydrodynamic diameter of the synthesized nanoparticles can be discovered using dynamic light scattering (DLS) also known as photon correlation spectroscopy (PCS). Herein bio-inspired synthesis involves formation of poly-dispersed nanoparticles. The hydrodynamic diameter is measured using Malvern Instruments Ltd. The DLS is represented in Fig. 11 which reveals the hydrodynamic diameter of the synthesized AgNPs. When the light passed through the colloidal solution, it bombards on small particles and scatters in all possible directions (Rayleigh scattering). We observe a fluctuation in the intensity of light even if the incident light is monochromatic or laser. This fluctuation in intensity of light is due to tiny molecules in solution which continuously undergoes Brownian motion. As a result of the Brownian motion of a particle, the dimension of the particle can be determined. DLS assumes that all particles are spherical in nature. The DLS results are complemented by the images provided by AFM and SEM. DLS showed the average particle size of as-synthesized AgNPs are 296.2 nm. The particles are made up by biological way thus the particle size of nanoparticles cannot be controlled and it is high in range. Electro kinetic potential of the sample reveals the dispersion stability of the colloidal solution. Higher values of electro kinetic potentials predict a more stable dispersion. In fact, electro kinetic potential analyzer is an important tool for understanding the state of the surface of the nanoparticle and predicting the long term stability of the nanoparticles. The zeta potential is an indication of the surface potential, and so determines the magnitude of the electric double layer repulsion. Normally, a value 40–60 mV indicates good stability of the nanoparticles.

Zeta potential shows the stability of the synthesized nanoparticles. It also shows the mobility of the nanoparticles. Zeta potential analysis of synthesized Ag nanoparticles shows incipient instability i.e. − 19.02 mV. According to zeta potential results, it was observed that the outer layer of Ag nanoparticles should possess negative charge. The figure shows three cycles reading of the stability and mobility of the nanoparticles. The zeta potential is an indication of the surface potential, and so determines the magnitude of the electrical double layer repulsion. The large positive and large negative value of zeta potential is required for stable dispersion.

## Ag nanoparticles as nano-catalyst

Reduction process is an important and fundamental of organic transformation in chemical synthesis and industrial chemistry. The description of a catalyst these days can be simply and ideally started with nanoparticles. In the current experimentation process, we have studied several organic transformation reactions using Ag nanoparticles as a nano-catalyst. Here we have tried to convert –NO_2_ group into –NH_2_ group using sodium borohydride as reducing agent. Actually sodium borohydride can reduce only the carbonyl group i.e. aldehydic (–CHO) or ketonic (–C=O) groups. However, our experimentation reveals that sodium borohydride can successfully reduce –NO_2_ functional group into –NH_2_ group in the presence of Ag nanoparticles. The progress of reaction is spectrophotometrically monitored^23,27,29–31,41^. The details of the studied reactions are as below.

### Nano-Ag catalyzed conversion of 4-nitrophenol into 4-aminophenol

**Figure 12:**
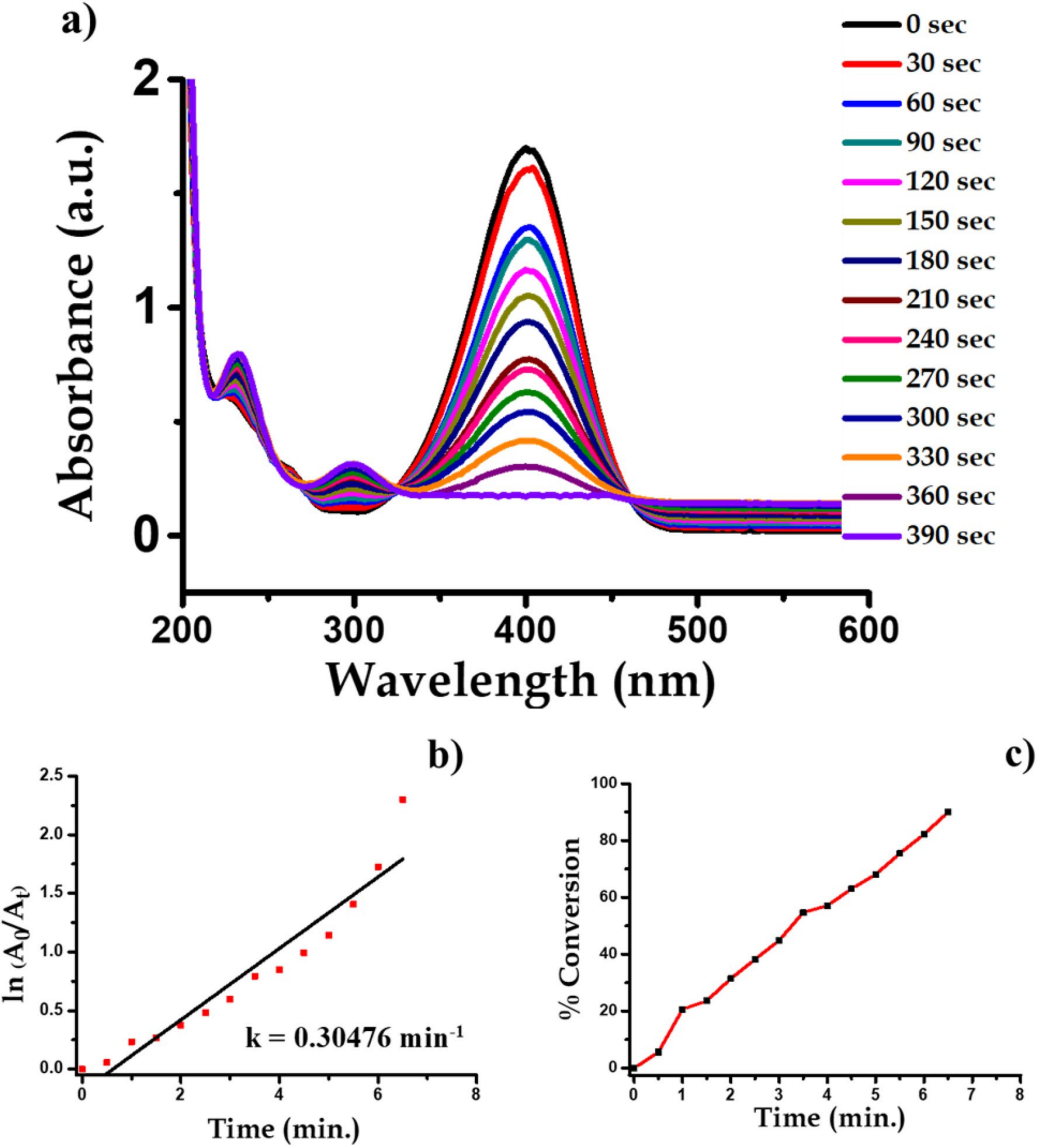
UV–visible spectra indicating (**a**) reduction of 4-nitrophenol to 4-aminophenol with time, (**b**) kinetics of catalytic reduction and (**c**) percentage conversion of 4-nitrophenol.

**Figure 13:**
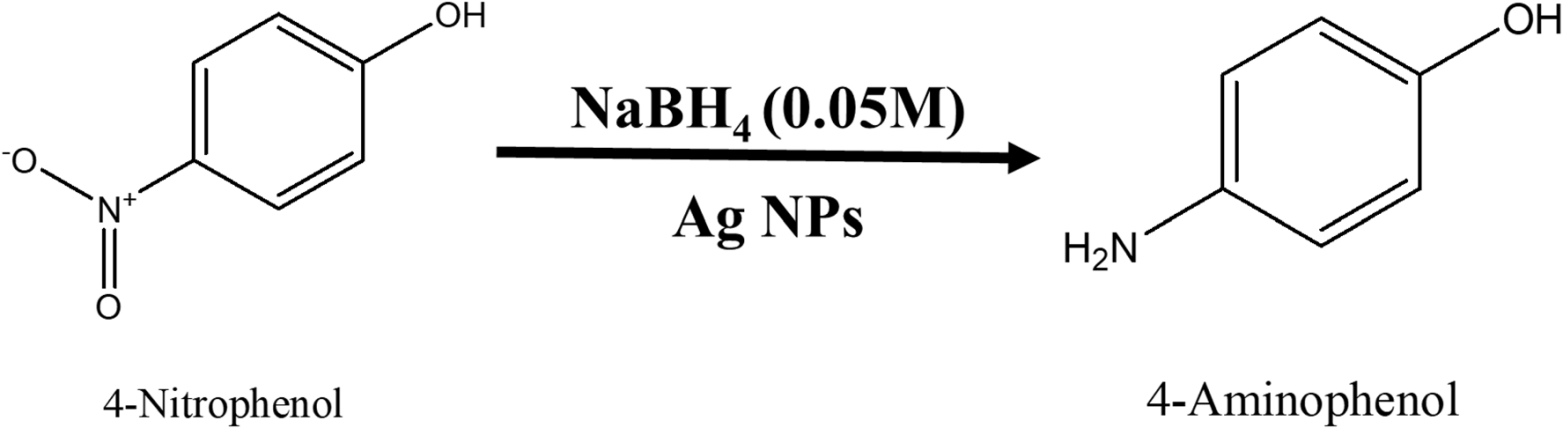
Schematic chemical reaction of conversion of 4-nitrophenol into 4-aminophenol.

For the current experimentation process, we purchased analytical grade 4-Nitrophenol (C_6_H_5_NO_3_) and Sodium Borohydride (NaBH_4_), from Sigma Aldrich. About 1 mL ice-cold solution of 0.05 M NaBH_4_ was taken in the quartz cuvette. Then, in the above solution about 1.5 mL, 0.1 mM 4-Nitrophenol solution was slowly added. To the reaction mixture, water suspension 200 μL (0.1 mg mL^−1^) Ag nanoparticles were added and we found that the conversion of 4-nitrophenol into 4-aminophenol takes place, which have been confirmed spectrphotometrcially. The absorption peak of –NO_2_ functional group is at wavelength 400 nm which decreases with progress of time whereas a new absorption at 300 nm makes its appearance which is responsible for –NH_2_ group. This indicates that complete conversion of 4-nitrophenol to 4-aminophenol take place within 390 s time interval. During this time about 95% of the reactant is converted into product. When we plot a graph of concentration verse time we get a straight line passing through origin. The progress of reaction is shown in Fig. 12. The conversion of 4-nitrophenol to 4-aminophenol with first-order reaction at a rate constant (k) is 0.30476 min^−1^. The reaction is shown in Fig. 13.

### Nano-Ag catalyzed conversion of 2-nitroaniline to 2-aminoaniline

**Figure 14:**
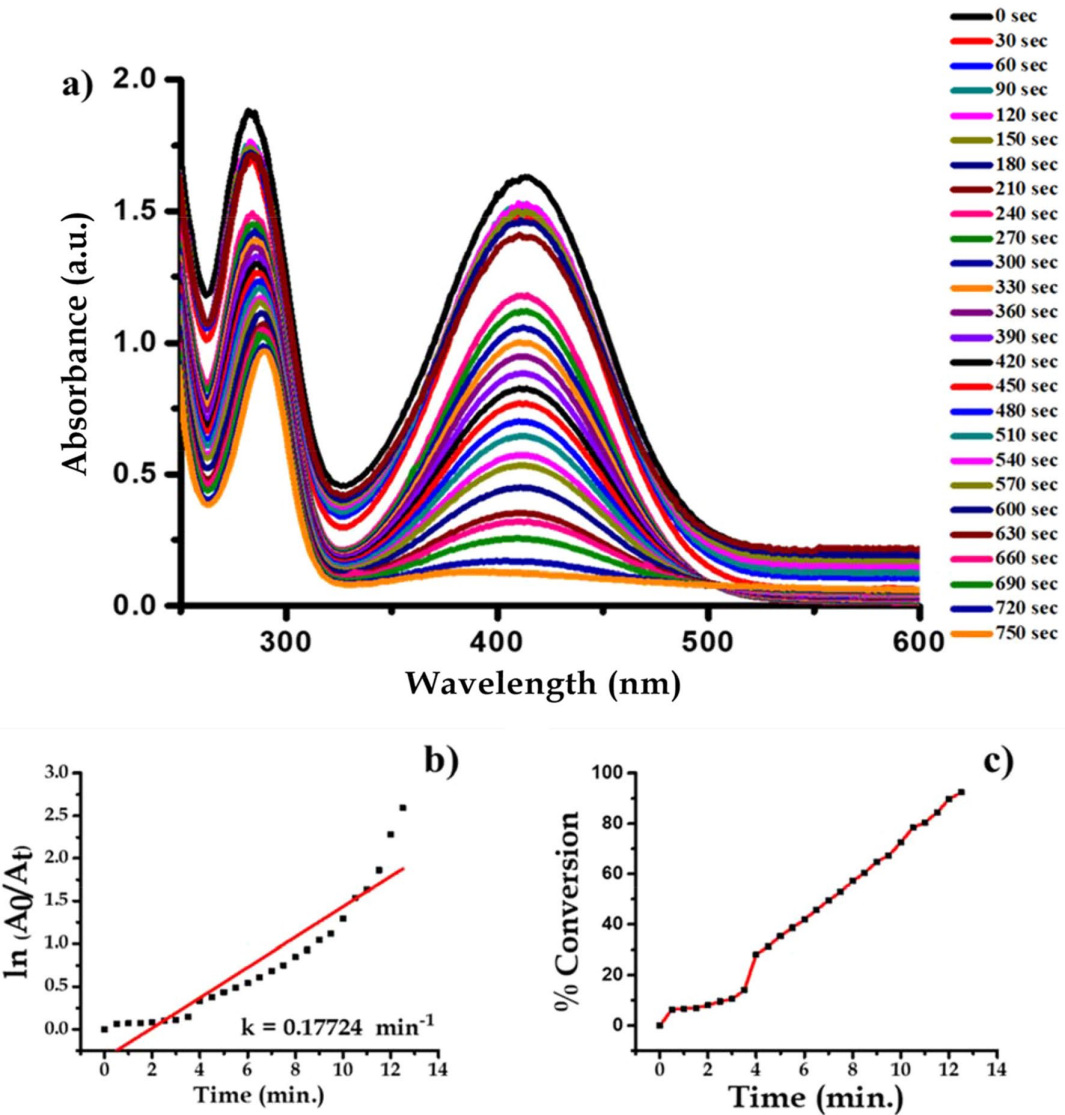
UV–Vis absorption spectra of (**a**) 2-nitroaniline, (**b**) kinetics of catalytic reduction and (**c**) percentage conversion of 2-nitroaniline.

**Figure 15:**
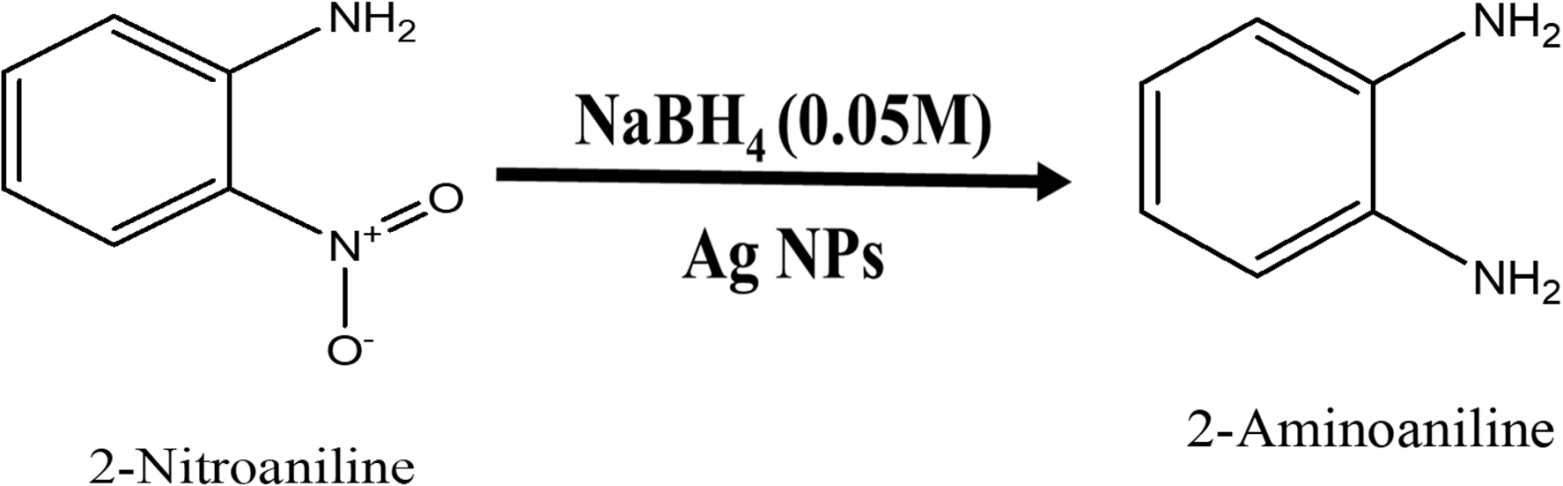
Schematic chemical reaction of conversion of 2-nitroaniline to 2-aminoaniline.

Analytical grade 2-nitroaniline (C_6_H_6_N_2_O_2_), Sodium Borohydride (NaBH_4_), were procured from Sigma Aldrich. About 1.5 mL ice-cold solution of 0.05 M NaBH_4_ was taken in the quartz cuvette. Then, in the above solution, about 1 mL 0.1 mM 2-Nitroaniline solutions were added drop-wise. To this reaction mixture water suspension of 200 μL (0.1 mg mL^−1^) Ag nanoparticles were added. We confirmed that the conversion of 2-Nitroaniline to 2-aminoaniline by the absorption peaks of reactant and product, spectrophotometrically. The absorption peaks responsible for –NO_2_ functional group is at wavelength 413 nm which decreases with progress of time. This indicates that complete conversion of 2-Nitroaniline to 2-aminoaniline take place within 750 s interval of time. During this time about 95% of the reactant is converted into product. The progress of reaction with a rate constant (k) is 0.17724 min^−1^ is shown in Fig. 14. The reaction is mentioned in Fig. 15.

### Nano-Ag catalyzed conversion of 3-nitroaniline to 3-aminoaniline

**Figure 16:**
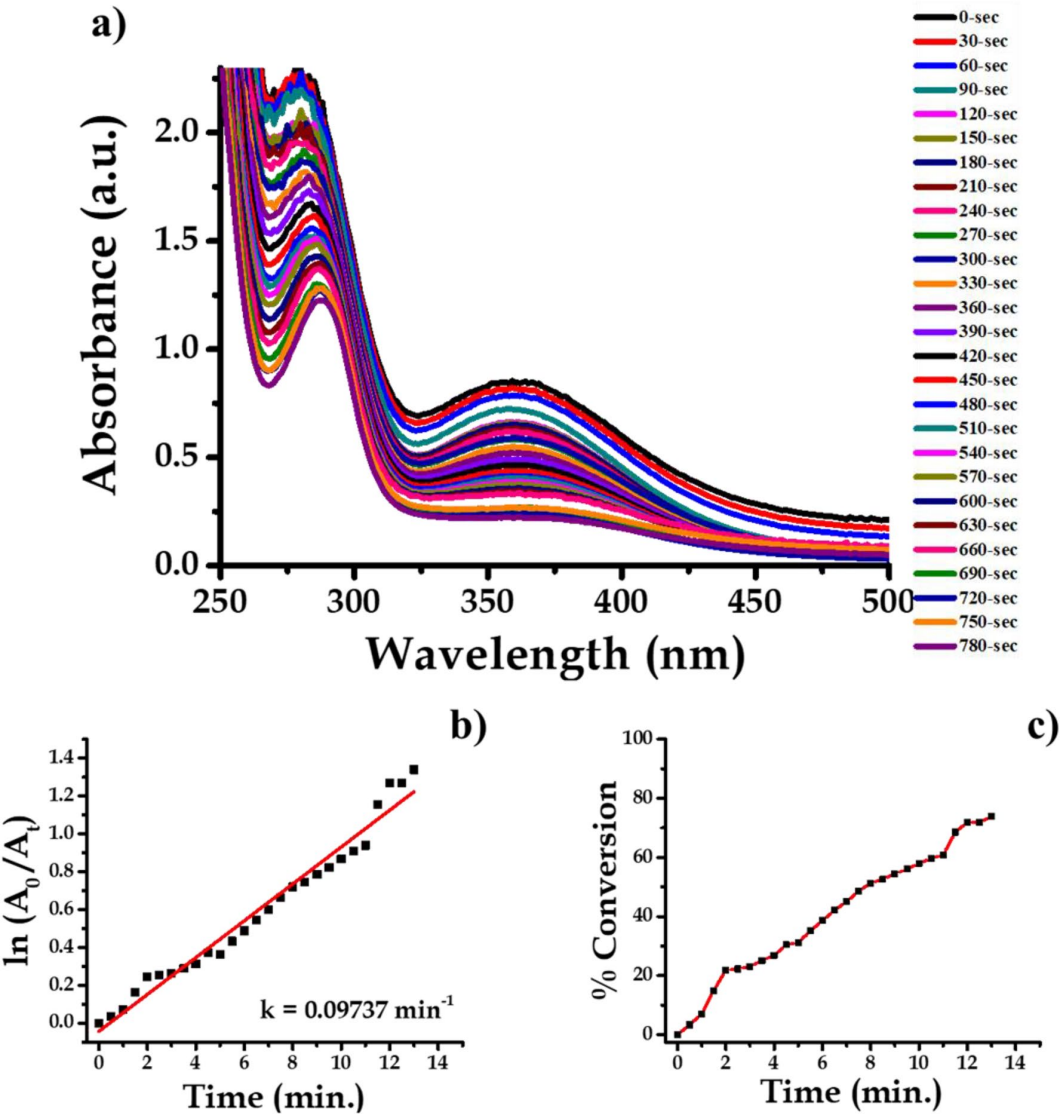
UV–Vis absorption spectra of (**a**) 3-nitroaniline, (**b**) kinetics of catalytic reduction and (**c**) percentage conversion of 3-nitroaniline.

**Figure 17:**
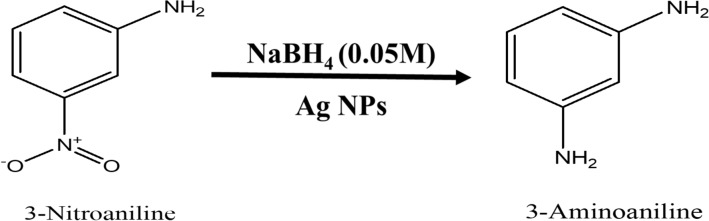
Schematic chemical reaction of conversion of 3-nitroaniline to 3-aminoaniline.

Analytical grade 3-Nitroaniline (C_6_H_6_N_2_O_2_), Sodium Borohydride (NaBH_4_), were procured from Sigma Aldrich. About 1 mL ice-cold solution of 0.05 M NaBH_4_ was taken in the quartz cuvette. Then, in the above solution about 1.5 mL 0.1 mM 3-Nitroaniline solutions was drop-wise added. To this reaction mixture water suspension of 200 μL (0.1 mg mL^−1^) Ag nanoparticles were added. The absorption peaks responsible for –NO_2_ functional group is at wavelength 363 nm which decreases with progress of time. This indicates that complete conversion of 3-Nitroaniline to 3-aminoaniline take place within 780 s time interval. During this time about 75% of the reactant is converted into product. When we plot a graph of concentration verse time we get a straight line passing through origin. The progress of reaction is shown in Fig. 16 with the first-order reaction with a rate constant (k) is 0.09737 min^−1^. The reaction is mentioned in the Fig. 17.

### Nano-Ag catalyzed conversion of 4-nitroaniline to 4-aminoaniline

**Figure 18:**
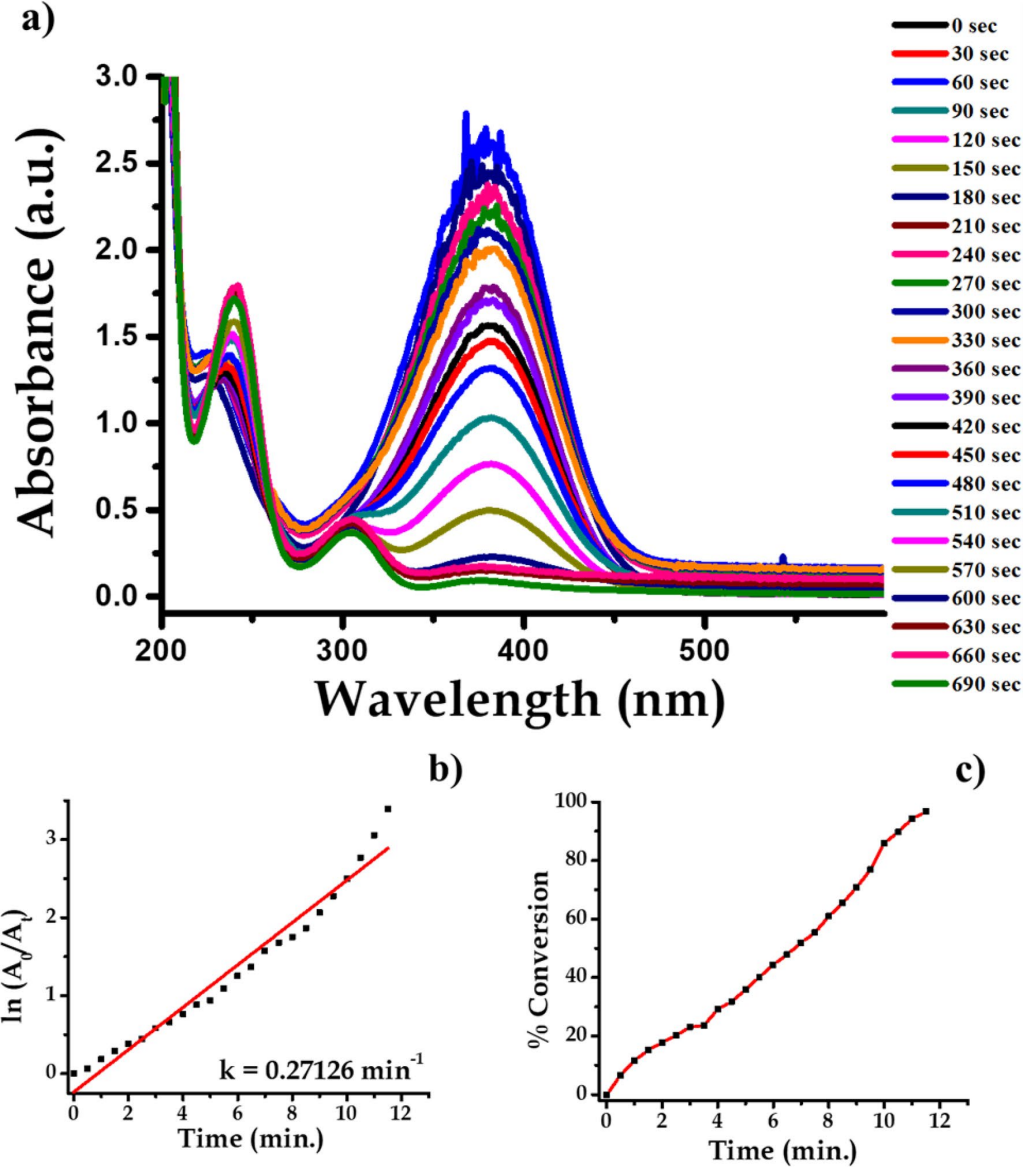
UV–Vis absorption spectra of (**a**) 4-nitroaniline, (**b**) kinetics of catalytic reduction and (**c**) percentage conversion of 4-nitroaniline.

**Figure 19:**
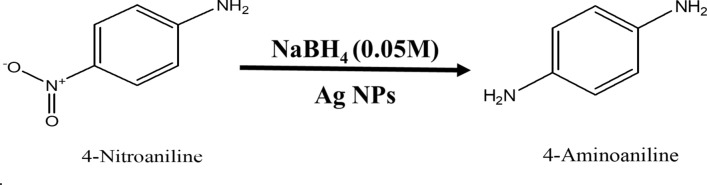
Schematic chemical reaction of conversion of 4-nitroaniline to 4-aminoaniline.

Analytical grade 4-Nitroaniline (C_6_H_6_N_2_O_2_), Sodium Borohydride (NaBH_4_), were procured. About 1.5 mL ice-cold solution of 0.05 M NaBH_4_ was taken in the quartz cuvette. Then, in the above solution about 0.5 mL 0.1 mM 4-Nitroaniline solutions was drop-wise added. To this reaction mixture water suspension of 200 μL (0.1 mg mL^−1^) Ag nanoparticles were added. The absorption peak responsible for –NO_2_ functional group is at wavelength 382 nm which decreases with progress of time whereas a new absorption at 280 nm makes its appearance which is responsible for –NH_2_ group. This indicates that complete conversion of 4-Nitroaniline to 4-aminoaniline take place within 690 s time interval. During this time about 98% of the reactant is converted into product. The progress of reaction is shown in Fig. 18 and reaction is shown in Fig. 19. This is an example of the first-order reaction with a rate constant (k) is 0.27126 min^−1^.

### Nano-Ag catalyzed dye degradation

Dye is an integral part which is used to impart color to materials. Textile industries heavily use natural and synthetic dye to color the fabric. The excess amount of dye is discharged which constitute major sources of water pollution. A dye is a high molecular weight organic compound. Such type of high molecular weight compounds is not easily degraded through natural process. Thus it contaminates surface water reservoir, soil and environment for long period which affects aquatic flora and fauna. Therefore, attempts have been made by researchers to degrade high molecular weight organic compounds into simple molecules. Herein Ag nanoparticles act as nano-catalyst for the degradation reactions of methylene blue and crystal violet using UV light. These two dyes methylene blue and crystal violet are selected for our study because they shows different colours in the oxidized and reduced forms and also their absorption maximum does not overlap with the SPR band of AgNPs^33,42–44^.

#### Nano-Ag catalyzed degradation of methylene blue (MB)

Methylene blue solution was prepared by dissolving 10 ppm of methylene blue (Methylthioninium Chloride) (C_16_H_18_ClN_3_S) in 80 mL of double-distilled water. In the methylene blue solution 100 mg synthesized Ag nanoparticles were added which behaves as heterogeneous catalyst. Then the beaker is wrapped with aluminum foil so as to avoid exposure of light. The solution in the beaker was rotated with magnetic needle. This results in adsorption of dye stuff on surface of nanoparticles. The reaction system was kept in darkness for 2 h. Then after 2 h reaction system was kept in light and absorption was measured at definite interval of time. The spectrophometric analysis was carried out both under ultraviolet light and visible light so as to investigate the efficiency of nanoparticles.

**Figure 20:**
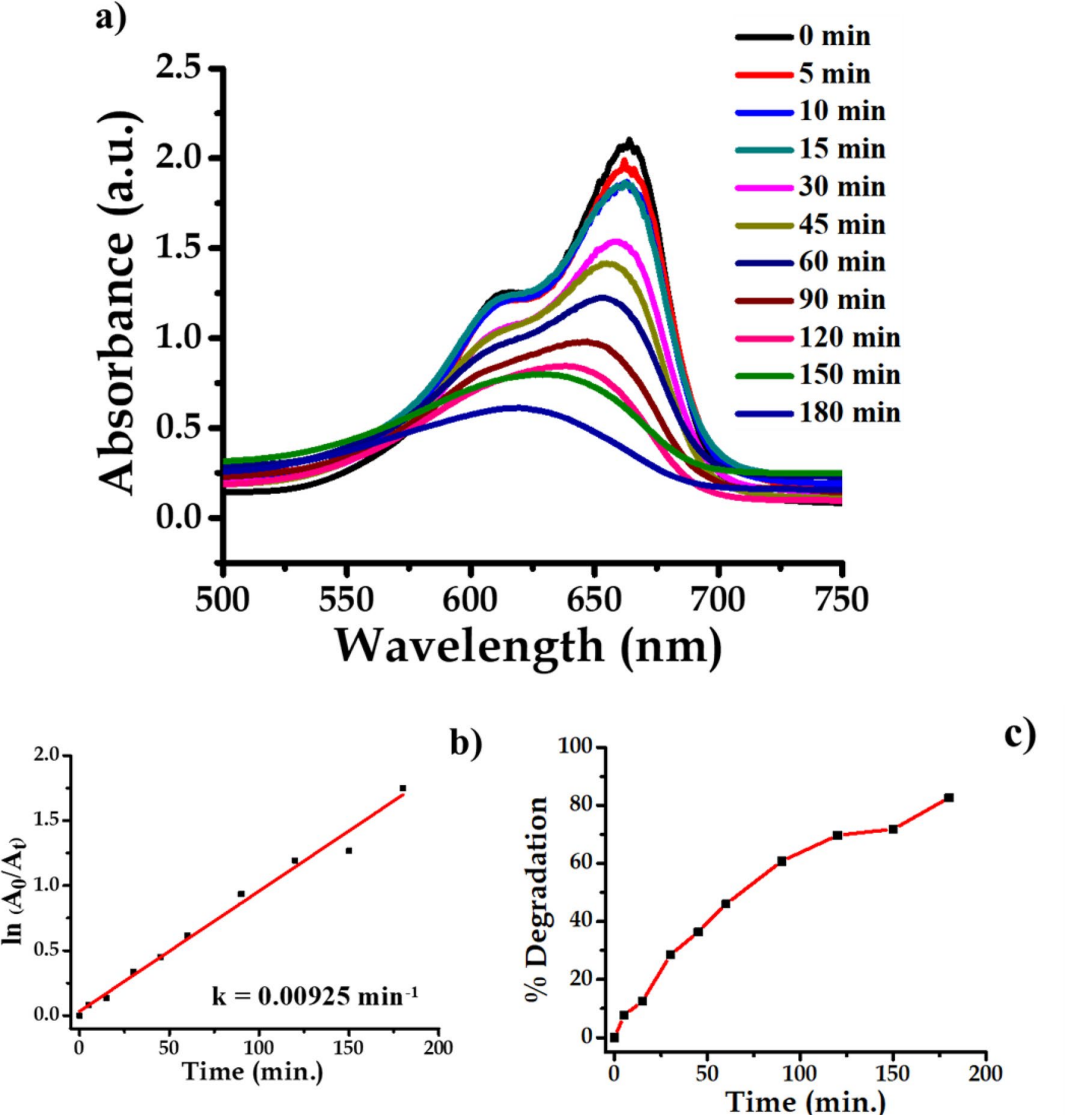
UV–Vis absorption spectra of (**a**) methylene blue, (**b**) kinetics of catalytic reduction and (**c**) percentage degradation of methylene blue.

The photocatalytic efficacy of AgNPs was determined both in UV radiation and visible light. Methylene blue was used as a test contaminant since it has been extensively used as an indicator for the photocatalytic activities owing to its absorption peaks in the visible range. The UV–Vis analysis reveals that about 80% degradation of dye takes place in 180 min. This is an example of first order kinetic reaction with rate constant 0.00925 min^−1^. The phocatalytic degradation of dye is spectrophotometrically monitored and shown in Fig. 20.

#### Nano-Ag catalyzed degradation of crystal violet (CV)

**Figure 21:**
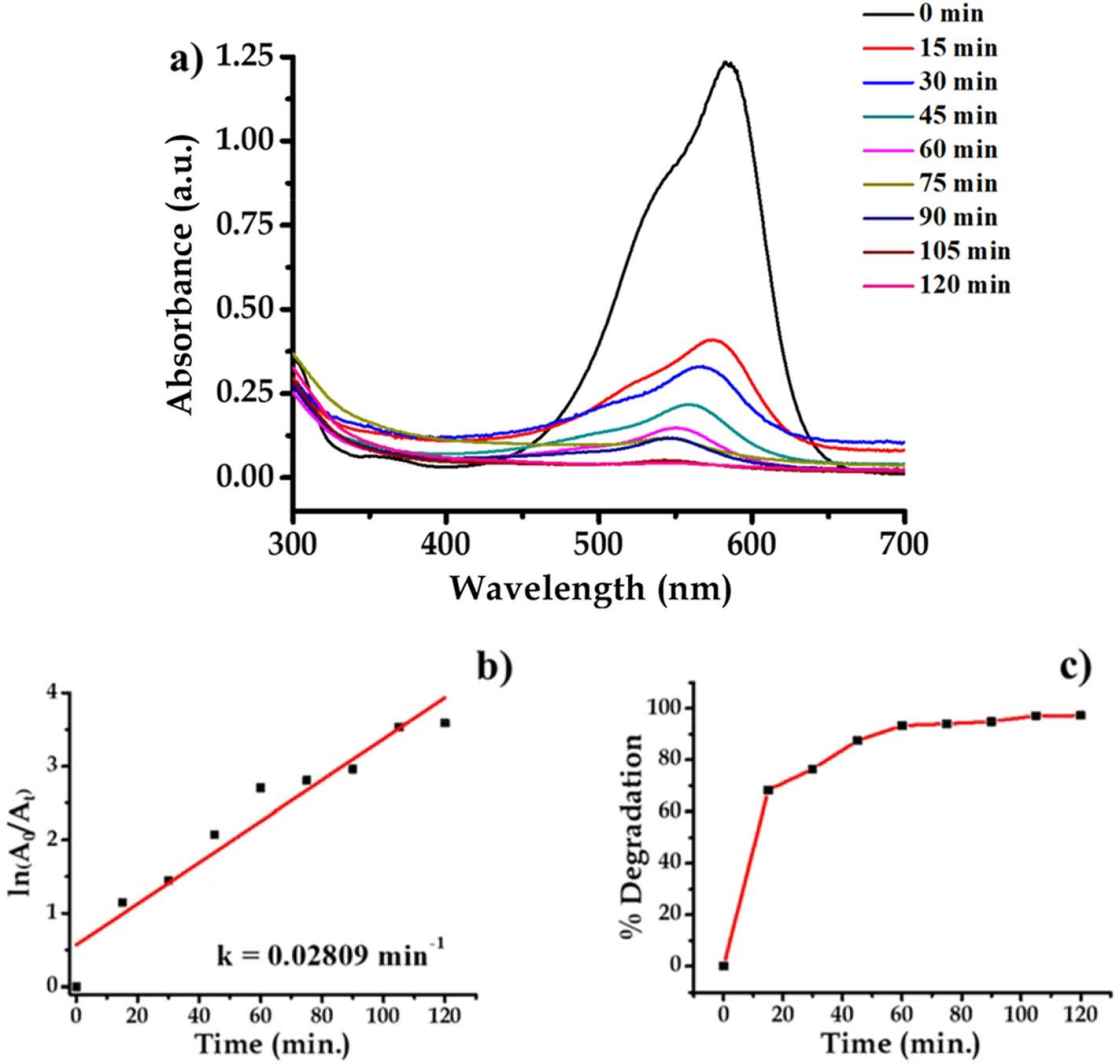
UV–Vis absorption spectra of (**a**) crystal violet, (**b**) kinetics of catalytic reduction and (**c**) percentage degradation of crystal violet.

Crystal violet or gentian violet is a triaryl methane dye. In fact, crystal violet is not only a dye but is a multi-applicative compound which finds use in bacterial staining, used as anti-bacterial, antifungal and antihelminthics medicine especially used as poultry medicine by veterinary doctors. When dissolved in water dye imparts blue–violet shade. Crystal violet solution was prepared by dissolving 10 ppm of crystal violet (Tris(4-(dimethylamino)phenyl) methylium chloride) (C_25_H_30_N_3_Cl) in 80 mL double distilled water. Then 100 mg Ag nanoparticle was dispersed. The beaker was completely wrapped with aluminum foil and kept on magnetic stirrer and rotated. The beaker was kept in darkness for 2 h. The evaluation was carried out both under ultraviolet radiation and visible light so as to investigate the efficiency of nanoparticles. The UV–Vis analysis reveals that about 75% degradation of dye takes place in 120 min. This is an example of first order kinetic reaction with rate constant 0.02809 min^−1^. The photocatalytic degradation of dye is spectrophotometrically monitored and shown in Fig. 21.

### Ethical statement

We all the authors hereby declare that all the ethical aspects have been taken into consideration while performing the experiment.

## Conclusion

Herein, we have successfully synthesized Ag nanoparticles using Indian cow (A-2) urine. As cow urine is used as reducing agent this method of synthesis is very much cost effective and environmentally benign. In fact, Ag nanoparticles are synthesized from the waste product alone. The bio-synthesized Ag nano-particles are potent catalyst for organic transformation reactions. The progress of organic transformation was monitored using spectrophotometer. The synthesized nanoparticles have been successfully used as a photocatalyst for degradation of hazardous organic dyes such as methylene blue and crystal violet.
